# Decoding the Role of CYP450 Enzymes in Metabolism and Disease: A Comprehensive Review

**DOI:** 10.3390/biomedicines12071467

**Published:** 2024-07-02

**Authors:** Basma Hossam Abdelmonem, Noha M. Abdelaal, Eman K. E. Anwer, Alaa A. Rashwan, Mohamed Ali Hussein, Yasmin F. Ahmed, Rana Khashana, Mireille M. Hanna, Anwar Abdelnaser

**Affiliations:** 1 Institute of Global Health and Human Ecology, School of Sciences and Engineering, The American University in Cairo, New Cairo 11835, Egypt; basmahosamabdelmonem@aucegypt.edu (B.H.A.); mohamed_hussein@aucegypt.edu (M.A.H.); yassmin.f.ahmed@aucegypt.edu (Y.F.A.); rkhashana@aucegypt.edu (R.K.); mireillehanna@aucegypt.edu (M.M.H.); 2 Department of Microbiology and Immunology, Faculty of Pharmacy, October University for Modern Sciences & Arts (MSA), Giza 12451, Egypt; 3 Biotechnology Graduate Program, School of Sciences and Engineering, The American University in Cairo, New Cairo 11835, Egypt; nohamabdelaal@aucegypt.edu (N.M.A.); emananwer@aucegypt.edu (E.K.E.A.); alaarashwan@aucegypt.edu (A.A.R.); 4 Department of Microbiology and Immunology, Faculty of Pharmacy, Modern University for Technology and Information, Cairo 4411601, Egypt

**Keywords:** CYP450, polymorphism, fatty acid metabolism, lipid metabolism, vitamin D metabolism, bile acids detoxification, hormone metabolism, PCOS, cancer, NAFLD, cirrhosis

## Abstract

Cytochrome P450 (CYP450) is a group of enzymes that play an essential role in Phase I metabolism, with 57 functional genes classified into 18 families in the human genome, of which the CYP1, CYP2, and CYP3 families are prominent. Beyond drug metabolism, CYP enzymes metabolize endogenous compounds such as lipids, proteins, and hormones to maintain physiological homeostasis. Thus, dysregulation of CYP450 enzymes can lead to different endocrine disorders. Moreover, CYP450 enzymes significantly contribute to fatty acid metabolism, cholesterol synthesis, and bile acid biosynthesis, impacting cellular physiology and disease pathogenesis. Their diverse functions emphasize their therapeutic potential in managing hypercholesterolemia and neurodegenerative diseases. Additionally, CYP450 enzymes are implicated in the onset and development of illnesses such as cancer, influencing chemotherapy outcomes. Assessment of CYP450 enzyme expression and activity aids in evaluating liver health state and differentiating between liver diseases, guiding therapeutic decisions, and optimizing drug efficacy. Understanding the roles of CYP450 enzymes and the clinical effect of their genetic polymorphisms is crucial for developing personalized therapeutic strategies and enhancing drug responses in diverse patient populations.

## 1. Introduction

Metabolism is an intricate network of biochemical reactions catalyzed by enzymes. Living organisms fuel their energy needs and synthesize new building blocks derived from nutrients essential for several vital processes in the cell, such as growth, development, and reproduction [1]. Drug metabolism is the biotransformation of drugs into inactive or active forms based on their chemical nature, either in the active form or as a prodrug [2,3]. For instance, the lipophilic properties of drugs allow them to remain in the body for prolonged periods, potentially leading to toxicity. In contrast, metabolizing them involves the enzymatic conversion of lipophilic properties to hydrophilic ones, enhancing their water solubility followed by excretion via urine or bile [4].

Drug biotransformation plays a pivotal role in the biosynthesis of crucial endogenous compounds, lowering xenobiotic toxicity and improving xenobiotic excretion. Therefore, mediating homeostasis and preventing toxic drug metabolite accumulation is essential. It is commonly divided into two Phases: I and II. In Phase I reactions, several processes, such as oxidation, reduction, and hydrolysis, are used to incorporate polar groups into the drug molecules, therefore facilitating their excretion from the body. On the other hand, in Phase II reactions, coupling of drug metabolites to more polar molecules involves the incorporation of several transferases, including uridine diphosphate (UDP)-glucuronosyltransferases, sulfotransferases, and glutathione S-transferases, subsequently enhancing water solubility properties of the drug metabolites and facilitate their excretion [5]. 

### 1.1. Pharmacogenomics: A Core Component of Personalized Medicine

It is a well-established fact in the literature that the population elicits a significant variation in their response to different xenobiotics (such as drugs) or endogenous substances produced within the body. Hence, this sheds light on the impact of varying drug responses on individuals’ health, either positively or negatively, which resulted in the evolution of the original concept of pharmacogenetics [6]. While pharmacogenetics focuses on a single genetic variation that affects the drug response, pharmacogenomics is a new and challenging area of research that focuses on the study of instantaneous multiple genetic variations that affect drug efficacy across the population. Further integration with other omics domains will pave the way for more personalized approaches and a deep understanding of the complex network of factors such as age, weight, environmental factors, and metabolism aberrations that affect drug response that subsequently will aid healthcare professionals in providing more tailored diagnosis and treatment recommendations for drugs and doses depending on the patient’s genetic makeup, better drug response, and lower drug-associated adverse effects [7,8,9,10]. 

Indeed, pharmacogenomics identifies genetic and epigenetic variations that affect drug responses, decreases drug-related adverse effects, reduces drug costs, and replaces the traditional “one-fits-all” treatment approaches. Owing to these advantages, pharmacogenomics has garnered significant attention from renowned worldwide scientific organizations to develop recommendations for pharmacogenomics for clinical implementation [10]. The research in this area focuses on exploring more effective therapeutic strategies for several diseases that harness a widespread variation in patients’ responses to different drugs, such as diabetes, immune system disorders, cardiovascular diseases, infectious diseases, cancer, and neuropsychiatric disorders, among other conditions [7,10].

### 1.2. Cytochrome P450 Enzymes: Their Function, Characteristics, and Role in Disease

Cytochrome P450 enzymes (CYP450s) belong to a superfamily of membrane-bound hemoproteins that play a central role in metabolizing both xenobiotics and endogenous compounds that are found in all kingdoms and believed to have originated from one common ancestor three billion years ago [11,12]. They are present in most body tissues and are essential in various physiological processes. Polymorphisms in CYP450 enzymes play a primary role in metabolism as well as several pathological conditions such as neoplastic development, negative psychological behavior, and other disorders. For instance, women with specific CYP1A1 polymorphisms might have increased susceptibility to genital malignancies. Additionally, CYP2C19*2, 2D6*4, 2D6*10, and 1A1*2A have been linked to developing squamous cell tumors in head and neck cancer. Conversely, the presence of CYP2D6*4 polymorphism has been linked to a protective effect against breast cancer (BC) [13,14]. 

Our review aims to uncover the functions, characteristics, and variation in CYP450 genes and their role in developing various diseases and treatment responses. We contemporarily emphasize the different CYP enzyme families and their substrates, the effect of CYP450 gene polymorphisms on enzyme activity, and treatment response. We will delineate the role of molecular techniques in detecting CYP450 polymorphisms and the involvement of CYP450 genes in endogenous metabolism (specifically hormone metabolism). Additionally, we will discuss the role of microbial CYP450 enzymes in fatty acid oxidation and potential therapeutic opportunities, its role in vitamin D activation, and how CYP450s are implicated in various diseases such as polycystic ovary syndrome (PCOS), adrenal insufficiency, liver diseases, and multiple cancers including breast, ovarian, endometrial, and hepatocellular carcinoma (HCC). Finally, we will shed light on the impact of CYP450s on chemotherapy and the expected future implementations.

## 2. CYP450 Enzyme Families and Their Substrates

The human genome has 57 CYP450 genes classified into families and subfamilies based on their similarity in the gene sequence. It is recognized that these genes are spread across 18 families [15]. Nevertheless, the inconsistencies in reporting the exact number of CYP450 subfamilies arise due to the exclusion of specific isoenzymes, such as CYP1A1, CYP1A2, and CYP1B1, from the isoenzyme count and considering them as three distinct subfamilies [4,16]. Nevertheless, Wrighton et al. delineate that CYP1A is a subfamily that only includes CYP1A1 and CYP1A2 [17]. Similarly, inconsistencies regarding the CYP2 family were reported. For instance, Zhao et al. and McKinnon et al. propose that CYP2A13, CYP2C19, and CYP2C18 might represent independent subfamilies rather than belonging to CYP2A and CYP2C subfamilies, respectively [4,16]. Zhao et al. argued against the commonly accepted notion that the CYP4 family has five subfamilies rather than six [4]. Studies reporting 43 as the number of CYP450 subfamilies are likely to advocate that CYP1A1 and CYP1A2 members as a single subfamily (CYP1A), except Zhao et al., who advocate that the CYP4 family contains only five members, not six [4,12,18,19]. On the other hand, those proponents for the 44 subfamilies are likely to count 3, 13, and 6 subfamilies for the CYP1, 2, and 4 families, respectively [20,21]. The subfamily classification of other CYP families has been discussed deeply elsewhere [4,17,20]. The 18 CYP families are split into well-known 41 subfamilies (CYP1A, 1B, 2A, 2B, 2C, 2D, 2E, 2F, 2J, 2R, 2S, 2U, 2W, 3A, 4A, 4B, 4F, 4V, 4X, 4Z, 5A, 7A, 7B, 8A, 8B, 11A, 11B, 17A, 19A, 20A, 21A, 24A, 26A, 26B, 26C, 27A, 27B, 27C, 39A, 46A, and 51A) [20,21]. There is a lack of consensus regarding the exact number of subfamilies within some CYP families. 

Regarding their nomenclature, CYP450 genes are named based on the homology of their amino acid sequences, following a number-letter-number sequence. They are denoted by the root symbol CYP and are further categorized into different families and subfamilies based on their sequence similarities. The family number is assigned to each gene (e.g., CYP1, CYP2), indicating a minimum amino acid sequence similarity of 40% within the same family [4,21,22]. Subsequently, a subfamily letter is appended to the family number (e.g., CYP1A, CYP2D), requiring a minimum amino acid sequence similarity of 55% within the subfamily. A specific number is assigned to differentiate between individual enzymes or isoforms within a subfamily (e.g., CYP1A1, CYP2D6). Notably, the last number in the gene name represents the order of discovery for that sequence [23,24,25]. 

### 2.1. CYP 450 Enzymes Functions, Mechanism, and Regulation 

The CYP450 enzymes are found in nearly 90 percent of the documented enzymatic bioreactions and play critical roles in overall human health, particularly xenobiotic metabolism, cellular metabolism, and drug response [4]. Like xenobiotic metabolism, they are also involved in endogenous compound metabolisms, such as bile acid, steroidal hormones, arachidonic acid, unsaturated fatty acids, eicosanoids, and leukotrienes, as well as their involvement in the biosynthesis of bile acid biosynthesis, steroid biosynthesis [4,12]. Indeed, they are oxidase catalysts and mainly carry out their function by contributing to Phase I metabolism that renders the metabolite more polar, therefore enhancing their water solubility and priming them for Phase II conjugation reactions [26,27,28]. In the Phase I reaction, CYP450 enzymes conjugate two oxygen molecules to their ferrous heme iron and cooperate with a redox partner to facilitate the oxidation process. In Phase I reaction, consequently, various chemical reactions such as reductive and oxidative dehalogenation, deamination, aromatic hydroxylation, dealkylation, aromatic hydroxylation, epoxidation, oxidative demethylation, S- and N-oxidation reactions, and isomerization, depending on CYP450 enzymes’ specificity and the type of substrate [29]. 

Several factors can regulate the expression of CYP450 enzymes, including genetic polymorphisms, induction or inhibition by drugs, cytokines, nuclear receptors, and transcription factors discussed extensively [30]. Nuclear receptors such as the farnesoid X receptor (FXR), the pregnane X receptor (PXR), and the constitutive androstane receptor (CAR) regulate the genetic transcription of various CYPs enzymes such as CYP7A1, CYP8B1, and CYP3A4 [31,32,33,34,35]. The epigenetic modification, including DNA methylation, histone modifications, and ncRNA regulation, significantly impacts CYP450 enzymes and mediates the intraindividual variations in xenobiotic metabolism, ontogeny, and treatment response (covered elsewhere [36,37]). They are often located in the hepatocytes’ mitochondria and smooth endoplasmic reticulum [4,11,21]. Even though they are primarily produced in hepatocytes, most extrahepatic organs (intestinal membranes, skin, brain, lung, and kidneys) express CYP450 enzymes to varying degrees, with the small intestine expressing the highest levels [12,21,38]. The rate of drug metabolism can vary among individuals due to genetic polymorphisms and the expression of CYP450 enzymes [4]. Also, external factors such as dietary habits, other drugs concurrent use, tobacco smoking, and alcohol consumption may influence the expression and functionality of these enzymes. Table 1 depicts each CYP450 enzyme family’s primary functions, some known substrates, and their significant role in metabolizing exogenous and endogenous compounds.

### 2.2. Effect of CYP450 Genes Polymorphisms on Enzyme Activity and Pharmacological Response

An individual’s metabolic capacity is determined by the combination of alleles they have inherited from their parents. These alleles can be classified as either wild-type or variant alleles. Wild-type alleles are considered the “standard” type and are most prevalent in the general population. On the other hand, variant alleles can lead to reduced or even negligible enzyme activity. Individuals who inherit two wild-type alleles typically have “standard” metabolism rates and are known as extensive metabolizers. In contrast, those who inherit two variant alleles usually have little to no enzyme activity, making them poor metabolizers. Individuals who inherit one of each type of allele have reduced enzymatic activity and are classified as intermediate metabolizers. In some cases, gene duplication or amplification can result in more than two copies of wild-type alleles, leading to higher-than-normal enzyme activity impacting drug metabolism and response. These individuals are referred to as ultrarapid metabolizers [12,17].

More than 2000 mutations have been discovered in CYP450 genes, with certain types of polymorphisms having a tremendous impact on enzyme activity [17]. Hence, understanding the kinds of present polymorphisms in these genes is crucial for predicting individual drug responses and optimizing drug therapy. Different types of CYP450 genetic polymorphisms include single nucleotide polymorphisms (SNPs), premature stop codon, variable number tandem repeats (VNTRs), gene deletions, and copy number variations (CNVs). Examining these specific variations and their impact on enzyme activity and drug metabolism can deepen our understanding of the intricate relationship between genetic polymorphisms and pharmacological response. 

#### 2.2.1. Single Nucleotide Polymorphisms

SNPs are a single base substitution with several million SNPs identified, and novel SNPs continue to be discovered. Some SNPs lie outside the protein-coding regions, while others lie within the coding regions of genes. Thus, this may or may not alter the protein synthesis. It is worth noting that SNPs are different from mutations. While SNPs are characterized by a frequency of at least 1% in the population, mutations refer to genetic changes that occur at a lower frequency [40]. Analysis of the SNPs identified by text mining showed that they were primarily found within three polymorphic CYP450s, including CYP2D6 (with 114 SNPs), CYP2A6 (with 68 SNPs), and CYP2B6 (with 57 SNPs). This was observed across all ethnic groups [13]. The investigation only considered the known frequency of nucleotide changes to determine the magnitude of SNPs within CYP450. Despite significant variability in the capacity for CYP3A4-mediated drug metabolism among individuals, the genetic variants identified so far can’t explain this variability, even though 20 distinct alleles have been recognized [41]. SNPs can be divided into synonymous and non-synonymous SNPs. Synonymous polymorphisms occur when a change in the DNA sequence does not affect the protein’s amino acid sequence due to the genetic code’s redundancy. Synonymous SNPs were previously commonly regarded as silent mutations [42]. However, nowadays, our understanding of these SNPs has evolved; recent studies have shown that synonymous SNPs can affect mRNA splicing, stability, and structure, as well as protein folding, which can significantly impact protein function [42,43]. 

To illustrate, synonymous SNPs can affect mRNA secondary structure, impacting translation efficiency and protein folding. Additionally, they can alter codon usage bias, potentially affecting protein synthesis rates and protein-protein interactions. Furthermore, synonymous SNPs can modulate mRNA stability and degradation rates, leading to changes in protein expression levels [13]. Non-synonymous polymorphisms, on the other hand, result in a change in the amino acid sequence of a protein, which can lead to altered protein structure and function. This, in turn, can have various effects, ranging from mild to severe, affecting an individual’s response to medications [44]. It is important to note that several non-synonymous polymorphisms in CYP450 enzymes, each with implications for drug metabolism and personalized medicine [13]. Over 100 non-synonymous single amino acid substitutions have been reported for isoforms in CYP3A4 and CYP2C9 alone [45]. Moreover, The CYP2D6 gene, for instance, has multiple polymorphisms that can affect the metabolism of various drugs, including antidepressants and antipsychotics [46].

#### 2.2.2. Premature Stop Codons 

Premature stop codons can result in the degradation of the transcript through a process known as nonsense-mediated mRNA decay (NMD), or they can produce a protein that is missing critical functional domains [47]. Premature stop codons in the context of CYP450 can have several implications as they result in a premature termination of protein synthesis. The NMD surveillance mechanism helps protect cells from potentially harmful truncated proteins [47,48,49]. 

#### 2.2.3. Variable Number Tandem Repeat 

VNTRs are short DNA sequences repeated a variable number of times in tandem; these repeats are grouped and oriented in the same direction. Tandem repeats are generally present in non-coding DNA, and they can influence gene expression and protein function [50]. For example, a VNTR polymorphism in the *MIR137* gene has been associated with an increased risk of colon cancer [51]. In the context of the CYP450 enzymes, specifically CYP2E1, VNTR polymorphisms are found in the 5′-flanking region of the gene. This polymorphism has been studied for health risks, particularly in cancers associated with drinking and/or smoking [51,52].

#### 2.2.4. Gene Deletions

Gene deletions involve the absence of a segment of DNA, which can result in the loss of gene function. Deletions in CYP450 genes can lead to a complete lack of enzyme activity for those genes [53]. One specific example is the *CYP2D6* gene. Deletions of this gene can alter the number of copies present, affecting the activity of the CYP2D6 enzyme. It is important to note that CYP2D6 polymorphisms can significantly impact drug metabolism, with some variations having up to a 200-fold effect on drug metabolism [54].

#### 2.2.5. Copy Number Variants 

CNVs are significant structural variations in the genome that result in the cell having an abnormal number of copies of one or more sections of the DNA. CNVs can include gene duplications or deletions, significantly affecting gene expression and function [55]. In the context of CYP450 genes, CNVs can result in an increased number of wild-type alleles, leading to higher-than-normal enzyme activity. In other words, copy number variants can lead to individuals being rapid metabolizers [55]. For instance, particular CNVs in CYP450 genes, such as CYP2C19 and CYP2D6, have been associated with rapid metabolizer phenotypes. CYP2C19*17 is a variant allele associated with increased enzyme activity, and individuals carrying this allele are considered ultrarapid metabolizers (UM) [56]. Similarly, CYP2D6 exhibits gene duplication, and individuals with multiple copies of the *CYP2D6* gene may also exhibit an ultrarapid metabolizer phenotype [57].

### 2.3. The Role of Molecular Techniques in Detecting CYP450 Polymorphisms for Personalized Medicine

Various molecular techniques are used to detect CYP450 polymorphisms, which play a crucial role in personalized medicine. These techniques include DNA microarray technology, DNA sequencing, polymerase chain reaction (PCR), and next-generation sequencing [58].

#### 2.3.1. DNA Microarray Technology

DNA microarray technology has been used as a research tool and in clinical applications. Initially, microarrays were used for large-scale population studies, such as determining the association between specific gene changes and diseases like BC [59]. The Food and Drug Administration (FDA) has approved several testing kits that utilize microarray technology for CYP450 genes, including the AmpliChip CYP450 test, the first FDA-approved pharmacogenetic test of its kind [60]. Another recent example is the Genomadix Cube CYP2C19 test, cleared for marketing by the FDA in March 2023. The Genomadix Cube CYP2C19 system provides results with 99.1% accuracy, enabling the identification of a patient’s CYP2C19 *2, *3, and *17 genotypes using DNA obtained from a buccal swab sample. By analyzing these genotypes, clinicians can, within an hour, better determine the therapeutic strategy for drugs metabolized by the CYP450 2C19 enzyme. This personalized approach to drug selection and dosing can lead to improved treatment outcomes and reduced adverse effects in 10% of clinically used drugs, including anti-platelet medication, antidepressants, and proton pump inhibitors [30,61]. Another microarray test is the xTAG Cyp2d6 Kit V3, an FDA-cleared test used to detect variations in the *CYP2D6* gene. This gene is involved in the metabolism of many drugs, including Eliglustat. Eliglustat treats a rare genetic disorder called Gaucher disease. CYP2D6 genotyping is recommended before prescribing eliglustat, as it is contraindicated in CYP2D6 ultra-rapid metabolizers and undetermined individuals of CYP2D6. In other words, the patient’s CYP2D6 genotype is crucial in determining the recommended dosing of eliglustat. This personalized approach based on genetic information using a microarray technique improves patient outcomes and enhances safety [62].

#### 2.3.2. DNA Sequencing

DNA sequencing is considered the gold standard for identifying key CYP450 polymorphisms. Sequencing technology has advanced significantly over the years, enabling the analysis of the entire genome or specific regions of interest [63]. Whole genome sequencing provides a comprehensive view of an individual’s genetic profile, including CYP450 polymorphisms. It has been widely used in research and clinical settings to understand the genetic basis of diseases, guide treatment decisions, and personalized medicine. In the context of CYP450 genotyping, DNA sequencing can validate the results obtained from other genotyping methods, such as microarrays. While the mentioned microarrays provide a high-throughput approach to analyze multiple genetic variants simultaneously, including CYP450 polymorphisms, sequencing can provide a more detailed and accurate assessment of the genetic variations in an individual’s genome [12].

#### 2.3.3. Polymerase Chain Reaction 

PCR is a laboratory technique used to amplify specific segments of DNA. It is widely used in genetic research and medical diagnostics. It allows for the rapid production of millions to billions of copies of a particular region of DNA, which can then be studied in greater detail [64]. Various techniques and methods based on PCR have been developed to specifically target and analyze genetic variations in genes such as the CYP450 genes [65]. Techniques such as allele-specific PCR (AS-PCR) and real-time PCR (RT-PCR) methods have been developed to specifically target and analyze variations in the CYP450 genes. These techniques utilize PCR to amplify and detect specific genetic variations. One technique, AS-PCR, is based on single-nucleotide variations (SNVs) and can detect any mutation involving a single base change. This competitive multiplex PCR method has been genotyping human CYP2E1 since at least 1999 [66]. 

AS-PCR has proven to be a valuable tool in determining the genotype frequencies and allele distributions of CYP2E1 polymorphisms in different populations. It has been used to detect specific genetic variants and SNPs in the CYP2E1 gene, providing insights into the role of CYP2E1 genetic variations in disease susceptibility and drug metabolism. On the other hand, RT-PCR methods, such as TaqMan assays, utilize fluorescent probes to detect specific genetic variations. For example, a study used RT-PCR with mutation-specific TaqMan probes to detect common variant alleles in CYP2C9, CYP2C19, and CYP2D6 genes in elderly hemodialysis patients. By allowing for the specific detection of genetic variations in these genes, this technique provided valuable information for personalized medicine approaches in patient care [67]. Furthermore, in personalized medicine, determining a patient’s CYP2C9 genotype through RT-PCR testing is crucial in administering certain medications. Take Siponimod, for example. Siponimod is an FDA-approved medication for the treatment of multiple sclerosis. Before initiating therapy with Siponimod, it is necessary to determine the patient’s CYP2C9 genotype through RT-PCR testing. This genetic testing helps determine the individual’s candidacy for Siponimod and the appropriate maintenance dosage. In patients with a poor metabolizer genotype for CYP2C9, Siponimod is contraindicated. On the other hand, in patients with an intermediate metabolizer (IM) genotype, it is recommended to use 50% of the normal maintenance dose of Siponimod [66,67]. Traditional methods such as PCR-RFLP (polymerase chain reaction-restriction fragment length polymorphism) and AS-PCR have also been employed in CYP450 genotyping [68].

#### 2.3.4. Next-Generation Sequencing 

Advances in next-generation sequencing have facilitated the identification of many rare variants across the human pharmacogenomic landscape, contributing to the overall genetically encoded functional variability in CYP genes. These techniques allow for the identification of genetic variants associated with specific drug toxicities [69]. For example, NGS has been successfully used to detect genetic variations, including polymorphisms, in the highly polymorphic *CYP2D6* gene and the promoter (TA)7 TAA repeat polymorphism UDP glucuronosyltransferase (UGT) 1A1*28 [70]. However, the functional effects of some common alleles in CYP450 genes remain controversial, as studies have shown varied results in metabolism rates when analyzing CNVs and SNPs in these genes [71]. Nonetheless, NGS has revolutionized genetic analysis by providing high-throughput sequencing, lower sample input requirements, higher accuracy, and the ability to detect variants at lower allele frequencies than traditional Sanger sequencing. Overall, NGS has significantly advanced our understanding of CYP450 polymorphisms and their impact on drug metabolism and personalized medicine [72].

## 3. Endogenous Metabolism

### 3.1. Hormone Metabolism

Steroid hormones are a class of hormones derived from cholesterol and play essential roles in various physiological processes, including metabolism, development, reproduction, and stress response [73]. The biosynthesis and metabolism of steroid hormones comprise a series of biochemical reactions that occur primarily in the adrenal glands and the gonads [73]. However, some steroid hormones are produced in other tissues, such as the liver, skin, and brain. The major steroid hormones include cortisol, aldosterone, testosterone, estrogen, and progesterone [74]. 

Pregnenolone (a precursor of steroid hormones) undergoes metabolism along specific pathways to produce distinct classes of steroid hormones. The direction of these pathways varies based on the tissue and the presence of enzymes. In the adrenal cortex, pregnenolone is transformed into glucocorticoids, including cortisol, which regulates metabolism, immune responses, and stress response [74]. Additionally, mineralocorticoids like aldosterone, a hormone vital for maintaining electrolyte balance and blood pressure, are produced. In the gonads, pregnenolone is converted to androstenedione, which eventually becomes testosterone. Testosterone is the primary male sex hormone responsible for developing male reproductive tissues and secondary sexual characteristics. In the ovaries, pregnenolone is transformed into progesterone, which serves as the precursor for synthesizing estrogens, such as estradiol (E2) [74]. These hormones participate in the development and regulation of the female reproductive system.

During steroidogenesis, specific classes of enzymes play vital roles in steroid hormone synthesis, activation, and inactivation. The first class of enzymes, hydroxysteroid dehydrogenase (HSD), depends on NAD(P)H and NAD(P)+ co-factors for its function. Based on their structural fold, HSDs are classified into two enzyme superfamilies: short-chain dehydrogenases and Aldo-keto reductases. They are further subcategorized based on their origin and utilization of NADPH as an electron donor [74]. The second class of enzymes is the CYP450s. The roles of CYP450 isoenzymes in steroidogenesis are summarized in Table 2 [75].

Both enzyme families play a pivotal role in converting hydroxysteroids to their respective ketosteroids and vice versa, thereby regulating the steroid activity at specific steroid receptors. These enzymatic reactions are essential for maintaining appropriate steroid hormone levels and ensuring normal physiological functions. For instance, CYP17 (17α-hydroxylase/17,20-lyase) plays a key role in the conversion of pregnenolone and progesterone into 17α-hydroxy pregnenolone and 17α-hydroxyprogesterone, which are precursors for the synthesis of cortisol and adrenal androgens. CYP19 (aromatase) converts androgens, such as testosterone and androstenedione, into estrogens, including estradiol [75]. The significance of CYP450 lies in its role in maintaining hormonal balance and homeostasis within the body. These enzymes regulate steroid hormone synthesis and metabolism, preventing excessive accumulation or depletion [76]. 

#### 3.1.1. Mechanisms of CYP450 Enzymes in Steroid Hormone Regulation 

##### CYP11A1

CYP11A1 is primarily found in the adrenal gland cortex but is also expressed in the testis, ovary, placenta, thymus, and intestine. Its primary role is to break down cholesterol’s side chain to produce pregnenolone [77]. The process involves the initial hydroxylation at C22, leading to the production of 22R hydroxycholesterol. The active site repositions the side chain, resulting in the second hydroxylation at C20, producing 20R, 22R-dihydroxy cholesterol. This is followed by oxidative cleavage of the C20–C22 bond, which produces pregnenolone [78]. The enzyme receives electrons for these reactions from NADPH via a short electron transport chain comprising adrenodoxin reductase and adrenodoxin. Cholesterol transport to the inner mitochondrial membrane site of CYP11A1 action is regulated by StAR-related lipid transfer domain-3 (STARD3) or steroidogenic acute regulatory (StAR) protein. The activity of the StAR protein or STARD3 is elevated by both increasing protein synthesis and its posttranslational modifications, predominantly mediated by cAMP-dependent pathways activated by adrenocorticotropic hormone (ACTH), angiotensin II, or luteinizing hormone [78].

##### CYP17A1

CYP17A1 is found in the adrenal cortex, ovaries, placenta, and testes. It catalyzes the addition of a hydroxyl group (OH) to the carbon atom at position 17 of pregnenolone and progesterone, two precursor molecules in the steroid hormone biosynthesis pathway [79]. This step is necessary for the subsequent conversion of these molecules into cortisol (a glucocorticoid) and aldosterone (a mineralocorticoid), respectively [79]. CYP17A1 cleaves the carbon–carbon bond at positions 17 and 20 of the steroid substrate, forming androstenedione, a precursor for the synthesis of testosterone and estrogens. The activity of CYP17A1 is tightly regulated to maintain the balance of steroid hormone production. Various factors, including hormonal signals and genetic variations, influence it. For instance, the secretion of ACTH from the pituitary gland stimulates CYP17A1 activity in the adrenal glands, leading to increased production of cortisol [79]. Additionally, the enzyme is subject to feedback regulation by the downstream products of steroidogenesis. Microsomal P450 enzymes receive at least the first electron from CYP450 reductase, but the second electron can be delivered by either CYP450 reductase or cytochrome b5. While CYP17A1 hydroxylation activity is not affected by the presence or absence of cytochrome b5, CYP17A1 lyase activity is negligible in the absence of cytochrome b5 but is strongly stimulated in its presence [79]. The onset of adrenarche correlates with increased expression of cytochrome b5, which results in a corresponding increase in CYP17A1 lyase activity and, therefore, androgen production. Notably, cytochrome b5 expression only occurs in the zona reticularis of the adrenal gland, where CYP17A1 does both the hydroxylation and lyase reactions to produce androgens. Still, it is not expressed in the zona fasciculata, where CYP17A1 performs only hydroxylation to generate glucocorticoids [79].

##### CYP21A2

CYP21A2 plays a significant role in the 21-hydroxylation of progesterone and 17α-OH-progesterone to produce 11-deoxycorticosterone and 11-deoxycortisol, respectively. This process occurs primarily in the adrenal cortex. The human genome has two CYP21 genes, CYP21A1 (a pseudogene) and CYP21A2 (the functional gene), located on chromosome 6 in the major histocompatibility locus. During the reaction, the major 21-hydroxylase activities produce electron-deficient primary carbon radicals (-CH3 → -CH2•) [80].

##### CYP11B2

CYP11B2 is expressed in the zona glomerulosa, the adrenal gland’s outermost layer. The biosynthesis of aldosterone involves a three-reaction series catalyzed by CYP11B2 [81]. The process begins with 11-deoxycorticosterone 11β-hydroxylation, resulting in the formation of corticosterone. Corticosterone is then hydroxylated at position 18 to yield 18-hydroxycorticosterone, which is further oxidized at the same position to produce aldosterone. CYP11B2 is classified as a mitochondrial class I CYP450 and relies on both the NADPH-dependent flavoprotein adrenodoxin reductase and the soluble [2Fe-2S] iron-sulfur protein adrenodoxin for its biosynthetic activity [81]. The transfer of two electrons from NADPH to adrenodoxin reductase initiates aldosterone synthesis, followed by transferring one electron at a time from reduced adrenodoxin reductase to adrenodoxin. Adrenodoxin then diffuses to the membrane-bound CYP11B2 and transfers one electron at a time to its heme. Each of the three reactions catalyzed by CYP11B2 requires two electrons, thus requiring six electrons from adrenodoxin for the biosynthesis of one molecule of aldosterone [81]. 

##### CYP19A1

Aromatase is a key enzyme involved in the catalytic conversion of adrenal androgens, such as testosterone and androstenedione, to aromatic estrogens (estradiol and estrone) via three consecutive hydroxylation reactions. It consists of CYP450 hemoprotein and a flavoprotein, NADPH-CYP450 reductase (CPR), and is localized in the endoplasmic reticulum of estrogen-producing cells, which requires CPR for catalysis [82]. This complex catalyzes the conversion of steroidal C-19 androgens (androstenedione and testosterone) to C-18 estrogens (estrone and estradiol), the rate-limiting final step in synthesizing estrogens. In the first step, the androgen substrate is hydroxylated at C-19 to produce a 19-hydroxy intermediate. The 19-hydroxy intermediate is oxidized in the second step to produce a 19-oxo compound [63]. The last step in the aromatization reaction involves the oxidative cleavage of the C10-19 bond to produce estrogens (estrone and estradiol) and formic acid [82]. Aromatase activity is found in a variety of tissues in the body, including gonadal sites such as the ovaries in premenopausal women and the testes in men, and critical extragonadal sites such as the placenta, chondrocytes, and osteoblasts of bone, adipose tissue, muscle, and brain. Aromatase inhibitors are widely used to prevent the development and progression of estrogen-dependent breast cancers. Aromatase is vital in several biological processes, including breast development during puberty, uterine growth, bone maturation during adolescence, bone mineralization, lipid metabolism, and cardiovascular risk in adults. In pregnant women, it protects against the virilizing effects of fetal androgens [82].

### 3.2. Fatty Acids and Lipid Metabolism

#### 3.2.1. Overview of CYP450 Mechanism of Oxidation

CYP450 enzymes are monooxygenases that bind two oxygen molecules to their ferrous heme iron. One oxygen atom is introduced into the substrate, while the other is converted into water [29,83]. This reaction has various results depending on CYP450 enzymes’ specificity and the type of substrate they interact with, including epoxidation, oxidative demethylation, S- and N-oxidation reactions, reductive and oxidative dehalogenation, deamination, aromatic hydroxylation, dealkylation, aromatic hydroxylation, isomerization, and oxidation of aldehydes and alcohols [29]. In vivo, the functionality of most CYP450 enzymes relies on redox partner systems derived by NAD(P)H. An N-terminal transmembrane helix region in eukaryotes connects the endoplasmic reticulum-associated microsomal CYP450 enzymes to their natural redox partner, CPR. FMN and FAD/NADP(H) are the two critical domains of the enzyme CPR. NADPH supplies hydride ions as electrons to the FAD domain to be reduced to the hydroquinone state [29,84]. Then, these electrons are sent to the FMN domain, which then transports them individually to the heme iron of CYP450 to be reduced to the ferrous state, which makes it easier for oxygen to bind. The resultant ferric-superoxide species is then further reduced to a reactive ferric-peroxo. Later phases of the catalytic cycle consist of two sequential protonation steps that create a ferric-hydroperoxo species and a ferryl-oxo heme radical cation species upon dehydration. The latter is crucial to the chemistry of oxygen insertion that the CYP450 enzymes catalyze [85]. The majority of bacterial and archaeal P450 enzymes, as well as mitochondrial P450 enzymes, use a distinct redox partner system. In this system, two NAD(P)H electrons are first delivered to a ferredoxin reductase that binds FAD and then to ferredoxin itself, transferring two electrons to the CYP450 in a single step, initiating catalysis. Adrenodoxin reductase, an enzyme in mitochondria, transfers electrons to 2Fe–2S adrenodoxin [29,86].

#### 3.2.2. Role of Microbial CYP450 in Fatty Acid Oxidation and the Potential Therapeutic Opportunities

*Saccharomyces cerevisiae* (*S. cerevisiae*) has been associated with various infections, including vaginitis skin and systemic bloodstream infections [87]. The sterol 14alpha-demethylase enzyme (*CYP51*) is essential in the ergosterol biosynthesis pathway. Ergosterol is a primary constituent in fungal membranes, contributing to various cellular functions. So, inhibiting CYP51 disrupts ergosterol synthesis, altering ergosterol membrane fluidity and induction of fungal cell apoptosis. So, targeting CYP51 is a potential antifungal approach against *S. cerevisiae* [88]. Triazole compounds like ketoconazole bind to CYP51 heme iron and inhibit the enzyme activity [89,90].

*Mycobacterium tuberculosis* (*M. tuberculosis*) is a bacterium causing tuberculosis, a multi-systemic disease affecting the respiratory, musculoskeletal, lymphoreticular, central nervous, and reproductive systems and the gastrointestinal tract, skin, and liver [91]. It was reported that CYP124 oxidizes isoprenoids such as farnesol, farnesyl diphosphate, and geranylgeraniol, producing the corresponding ω-hydroxylated products. These products are essential for synthesizing important respiratory menaquinones, which play a role in *M. tuberculosis* survival [92]. Sulfated forms of these menaquinones have been reported to negatively regulate the immune response in mice infected with *M. tuberculosis* [93,94]. Additionally, *M. tuberculosis* exhibits various lipids with methyl branching on its cell surface, including sulfolipid-1, di- and polyacyl-treheloses, mannosyl-β-1-phosphomycoketide, phthiocerol dimycocerosate, and mycolic acids that can be used as carbon sources for bacterial energy production. All these findings suggest the potential target of CYP124 for treating *M. tuberculosis* infections [92]. A previous study reported the binding of azole drugs to CYP124 through coordination with the heme iron, suggesting the potential use of azole drugs for inhibiting *M. tuberculosis* infections [92]. Also, *CYP121A1* has been identified as an essential enzyme that catalyzes the phenol-coupling reaction required for the dipeptide dicyclotyrosine to generate mycocyclosin in *M. tuberculosis*. Previous studies reported *M. tuberculosis* growth inhibition upon the knockout of the *CYP121A1* gene, suggesting that this enzyme could be a potential target for inhibiting infections with *M. tuberculosis* [95]. Previous studies reported the ability of azoles to inhibit *M. tuberculosis* growth in vivo and in vitro. Econazole, clotrimazole, and miconazole exhibit stronger binding to CYP121A1, significantly contributing to the suppression of *M. tuberculosis* growth [96].

*Pseudomonas aeruginosa* (*P. aeruginosa*) is an opportunistic bacterium that causes chronic lung infections. It mainly uses the phosphatidylcholine component of the host cellular membrane as a nutrient source to colonize the lung, leading to airway blockage and damage to the surface of the lung epithelial cell [97]. In brief, *P. aeruginosa* produces lipases and phospholipase enzymes that break down phosphatidylcholine to release fatty acids, which undergo further breakdown via the β-oxidation cycle to produce energy utilized for *P. aeruginosa* cellular processes. CYP450 enzymes are not directly involved in the β-oxidation cycle but in hydroxylating the produced fatty acids to be degraded through the β-oxidation cycle [98]. In a previous study, CYP168A1 from *P. aeruginosa* was characterized for its ability to catalyze the hydroxylation of some saturated fatty acids, including myristic, palmitic, and stearic acids and other unsaturated fatty acids, including palmitoleic, oleic, and linoleic acids, suggesting that CYP168A1 is a potential target for treating infections with *P. aeruginosa*. After testing miconazole and voriconazole, they did not affect the catalytic activity of *P. aeruginosa* CYP168A1 in the presence of oleic acid, suggesting that once the substrate is bound, these azoles do not easily displace it, and the currently available azoles would need modification to effectively target CYP168A1 for inhibiting *P. aeruginosa* [99].

#### 3.2.3. Role of CYP450 in Polyunsaturated Fatty Acid Metabolism

CYP450 enzymes are essential in metabolizing polyunsaturated fatty acids (PUFAs), forming either hydroxy-PUFAs via hydroxylase activity or epoxy (EP)-PUFAs via epoxygenase activity. The specific CYP enzyme and the type of PUFA substrate determine the final product formed. The B-C loop within each CYP enzyme’s structure significantly influences its catalytic properties, regio/stereo-selectivity, and overall structure, affecting the reaction type and the prevalence of specific regio/stereoisomer products [100,101,102,103,104].

AA is mainly produced by CYP2B, 2C, and 2J subfamilies (also called AA epoxygenase) as Ep-PUFAs, whereas 1A, 4A, and 4F subfamilies (also called AA hydroxylase) mainly create ω and ω-1 hydroxylated AA [103]. There is regio- and stereo-selectivity in both CYP families. For instance, four regioisomers of epoxyeicosatrienoic acids (EETs) may be produced from AA by the CYP2C and 2J subfamilies; these regioisomers can exist as the S, R- or the R, S stereoisomer. The various CYP isoforms differ in their regio- and stereo-selectivities. For instance, CYP2J2 has limited regio-selectivity and produces all four regioisomers of EETs, but CYP2C8 primarily metabolizes AA to 11,12- and 14,15-EET with excellent regio/stereo-selectivity [103,105]. Comparably, several EET products with excellent stereo-selectivity are produced by CYP2C23 and CYP2C44 [100]. Although the CYP subfamilies involved in the linoleic acid (LA) metabolism have not been thoroughly investigated, data indicates that all CYP isoforms can efficiently metabolize LA. For instance, CYP2C9 functions as the principal LA monooxygenase in the human liver, generating 9,10- and 12,13-epoxyoctadecamonoenic acids (EpOMEs) [82]. Other CYP isoforms, such as CYP2C8, -19; CYP2J2, -3, -5, -9; CYP1A2; and CYP3A4, that metabolize AA can also metabolize LA. Remarkably, when switching from AA to LA, the same CYP isoform may show distinct preferences when functioning as an epoxygenase or hydroxylase [100].

Similarly, eicosapentaenoic acid (EPA) and docosahexaenoic acid (DHA) can serve as substrates for various CYP isoforms in humans, rats, and mice [106]. For example, CYP2C8, 9, 18, 19, and CYP2J2 can epoxidize both EPA and DHA [107,108]. Furthermore, CYP2C isoforms exhibit similar catalytic activities for EPA and DHA compared to AA, while CYP2J2 elicits significantly higher rates in metabolizing EPA and DHA than AA. These enzymes also demonstrate different regio-selectivity for EPA and DHA [107,109,110]. CYP450 hydroxylate PUFAs in a regio-selective manner, similar to epoxidation activity. The primary products of the CYP4A and 4F subfamilies’ hydroxylation of AA at the terminal methyl group are 20-hydroxyeicosatetraenoic acid (20-HETE), whereas 19-HETE is the minor product. In contrast, AA is mainly metabolized by CYP1A1, CYP1A2, and CYP2E1 through ω-1 hydroxylase activity, with 19-HETE being the primary byproduct. In addition, various CYP hydroxylases, including mouse CYP 4A12 and rat CYP 4A1, may efficiently hydroxylate EPA and DHA [100,111]. These enzymes include human CYP4A11, CYP4F2, CYP4F3A, and CYP4F3B. When the substrate changes from AA to EPA or DHA, these enzymes exhibit altered regioselectivity and reactivity. For example, CYP4A1 hydroxylates AA principally to give 20- and 19-HETE while epoxidizes and hydroxylates EPA to a large yield of 17,18-EEQ and 19-HEPE [100,111].

#### 3.2.4. Role of CYP450 Enzymes in Cholesterol, Endogenous Toxins (Bile Acids), and Their Associated Diseases

Bile acid biosynthesis regulates the elimination of cholesterol from the body. This process involves seventeen highly regulated enzymes to maintain cholesterol balance and facilitate intestinal emulsification. When the body has excess cholesterol, excess bile acids suppress further synthesis, whereas low bile acid levels increase cholesterol synthesis [100]. Briefly, the metabolic pathway of cholesterol occurs via two pathways: the classic and alternative pathways. The classical pathway mainly eliminates 400–600 mg of cholesterol daily, primarily initiated by CYP7A1, and contributes to 90% of human bile acids synthesis. The key players CYP450s in this major pathway are CYP7A1 and CYP8B1 [112,113]. The main bile acids products in this pathway are the primary bile acids: cholic acid (CA) and chenodeoxycholic acid (CDCA). Cholesterol 7 alpha-hydroxylase (CYP7A1) is the rate-limiting enzyme in the classic pathway that converts cholesterol to 7 alpha-hydroxycholesterol [114]. As an intermediate product from multistep reactions by some intermediate enzymes, 3 beta-hydroxysteroid dehydrogenase (C4) is produced. It is converted to the primary bile acid, cholic acid (CA), by the sterol 12 alpha-hydroxylase (CYP8B1) [114]. The second primary bile acid in this pathway is CDCA, which could be produced directly from C4 without CYP8B1 [114]. In the same pathway, sterol 27-hydroxylase (CYP27A1) has a role in the production of CDCA. Although CYP27A1 has a role in synthesizing CDCA, it does not have a significant role in the classic pathway. Its role is more prominent in the alternative pathway [115]. The importance of CYP7A1 is highlighted when individuals lacking this enzyme activity due to CYP7A1 mutations exhibit high plasma cholesterol levels and become resistant to statin treatment, leading to elevated triglyceride levels and prone to conditions like gallstone disease [100].

For instance, in a previous study conducted in 2018, researchers analyzed lipid levels and examined eight rare non-synonymous mutations and two common variants (rs2081687 and rs3808607) in CYP7A1 among 100,149 individuals. They also investigated whether weighted allele scores for rs2081687 and rs3808607 were associated with increased plasma levels of low-density lipoprotein (LDL) cholesterol and whether these abnormal levels correlate with elevated risks of myocardial infarction and symptomatic gallstone disease. The results revealed that higher CYP7A1 allele counts were associated with a 12% increase in LDL cholesterol compared with the lowest allele count, leading to increased risks of myocardial infarction and gallstone disease [116]. Enhancing CYP7A1 activity achieved, for example, through bile acid-binding resins like cholestyramine, can lower cholesterol levels, even though with limited use due to tolerability issues. CYP7A1 activity varies among individuals, influenced by genetics, diet, age, and alcohol consumption. Thus, tailoring personalized approaches by screening the CYP7A1 genotype and enzyme activity is crucial for effective cholesterol management [100].

The alternative pathway is the second way for bile acid synthesis, contributing to 10% of the total bile acids. The main CYP450s in the alternative pathway are CYP27A1 and oxysterol 7 alpha-hydroxylase (CYP7B1) [117]. This pathway activity is increased when the classical pathway is negatively affected in liver injury or cirrhosis, converting cholesterol to 27-hydroxycholesterol [118]. 27-hydroxycholesterol is further metabolized by the hydroxylation activity of CYP7B1 to 3 beta, 7 alpha-dihydroxy-5-cholesterol acid, and eventually, it is converted into CDCA. Hence, the primary bile acid compounds are mainly generated via the previously stated pathways in the liver; however, the secondary bile acids are produced primarily in the intestine with the help of bacterial enzymes [118]. A bacterial enzyme called 7 alpha-hydroxylase converts CA and CDCA into deoxycholic acid (DCA) and LCA, respectively [118].

The secondary metabolites, DCA and LCA, are distinguished by their hydrophobicity and toxicity, and their detoxification is crucially required to prevent them from accumulating in the liver and eventually initiating liver disease [119]. Furthermore, the detoxification of these compounds is mainly catalyzed by three CYP450 enzymes, the CYP3A4, CYP2B, and CYP2C subfamilies. These CYP450 enzymes are responsible for converting the hydrophobic and highly toxic LCA and DCA into hydrophilic and less toxic hyocholic acid (HCA) and ursodeoxycholic acid (UDCA), respectively [120,121,122].

Although mutations in CYP27A1 lead to cerebrotendinous xanthomatosis (CTX), a disease characterized by cholesterol accumulation and premature atherosclerosis, the regulatory role of CYP27A1 remains uncertain. While bile acids influence CYP27A1 transcription, interindividual variability in enzyme activity appears less evident [100]. Furthermore, a case report in 2023 of a 60-year-old female presented with alterations in behavior and personality, hyperlipidemia, and elevated serum levels of cholestanol (12.4 mg/L; normal value ≤ 6.0), and, when undergoing genetic testing, revealed a pathogenic variant in the CYP27A1 gene, suggesting a diagnosis of CTX [123]. In the central nervous system, local cholesterol synthesis predominates due to the impermeability of the blood–brain barrier to circulating cholesterol [100]. CYP46A1, primarily expressed in neurons, hydroxylates cholesterol at position 24, facilitating its removal from the brain. Dysregulation of CYP46A1 is implicated in Alzheimer’s disease, although this correlation requires further elucidation. Moreover, CYP46A1′s product, 24(S)-hydroxycholesterol, is an endogenous ligand for liver X receptors (LXR), modulating cholesterol homeostasis and potentially influencing neurodegeneration progression. Recent structural studies of CYP46A1 suggest its conformational flexibility and potential inhibition by certain drugs, highlighting its pharmacological relevance beyond cholesterol metabolism [100].

#### 3.2.5. CYP450 Enzymes and Bile Acids-Binding Nuclear Receptors

Bile acids, including the primary and secondary compounds, are ligands for some nuclear receptors (NRs) that undergo conformational change upon bile acid binding and, therefore, induce some transcriptional regulations for some genes to regulate and optimize the bile acids and cholesterol in physiological amounts [32]. The nuclear receptors are classified according to their functional activity upon binding of bile acids into the following.

##### Nuclear Receptor Regulating Bile Acid Synthesis: FXR

The FXR is a nuclear receptor highly expressed and locally dominant in the liver. It has a high binding affinity for CDCA and mainly regulates the genetic transcription of CYP7A1 and CYP8B1 genes, known as primary bile acids synthesizing CYP450 enzymes [124]. FXR also regulates the transportation of bile acids; however, the focus in this part will be on the regulation of bile acid synthesis via FXR. The regulation of the bile acids biosynthesis process occurs upon the binding of bile acids to the FXR receptor that eventually recruits and activates another two small nuclear receptors: Small Heterodimer Partner (SHP) and Hepatocyte Nuclear Factor 4 alpha (HNF4 alpha) [125,126]. These nuclear receptors act as repressors that eventually bind to the promoter regions of both *CYP7A1* and *CYP8B1* genes by inhibiting the Liver Receptor Homolog 1 (LRH-1) (positive regulator of these genes) and, subsequently, inhibit their transcription [126].

Hence, this will decrease the production of bile acids from cholesterol. This regulatory cascade involving bile acids/FXR/SHP/HNF4 alpha or LRH-1/*CYP7A1* or *CYP8B1* could have a role in reducing the high amounts of bile acids accumulating in the liver. It may lead to cholestasis (a disease state in which there is an impairment of bile acids secretions of hepatocytes leading to an accumulation of bile acids) [127,128,129]. A research study investigated the role of FXR in bile acids homeostasis by examining the amounts of serum bile acids, triglycerides, and cholesterol in an ablated *FXR* gene in mice [124,130]. The study’s findings revealed elevated levels of serum bile acids and hepatic cholesterol in FXR-null mice, which was further proven by another that showed and confirmed the atherogenic effects of elevated harmful cholesterol levels (VLDL) in these *FXR-deficient* mice [33].

##### Nuclear Receptor Regulating Bile Acid Metabolism (Detoxification): PXR and CAR

The PXR is the second nuclear receptor, mainly located in the liver in high amounts [33]. This receptor has the highest affinity towards the toxic and lipophilic form of bile acid, LCA. Upon binding of LCA to PXR, a dimerization of PXR with RXR occurs, and together, they induce the expression of the *CYP3A4* gene via binding to its xenobiotic response element [131]. CYP3A4 converts LCA to 3-dehydro-LCA, hyodeoxycholic acid, and 1 beta-hydroxy-LCA. These forms are more hydrophilic and less toxic, as they could be easily eliminated and excreted in urine [131]. This detoxification reaction via PXR is usually considered a protection mechanism of reaction in the case of cholestasis [34]. This accumulation activates PXR and induces the mechanism discussed previously. Thus, the PXR-induced detoxification mechanism is considered a feedback mechanism of action to facilitate the detoxification and excretion of accumulated toxic forms of bile acids. The CAR is nearly like the PXR receptor in inhibiting the accumulation of bile acids in cholestasis [132]. However, CAR receptors do not bind to bile acids directly as they bind to bile acid toxic metabolites in the case of cholestasis [133]. Both CAR and PXR receptors induce the expression of the CYP3A4 enzyme to reduce the toxicity of these accumulated bile acids [132].

The FXR receptor is involved in bile acid homeostasis because it represses the expression of *CYP7A1* to decrease bile acid synthesis. The opposite case could happen if a non-functioning *FXR* gene demonstrates its inability to bind to its high-affinity substrates (bile acids) and negatively affects the repression of the bile acids synthesis process through a continuous synthesis by CYP7A1 [134]. This abnormal condition could lead to cholestasis or enhance the pathological conditions of cholestasis by increasing the accumulated amount of bile acids in the liver. As a result, this accumulation could lead to increased exposure of the hepatocytes to the toxic forms of bile acids, which eventually progress to fibrosis, cirrhosis, and HCC later in the disease [127]. Thus, a nuclear receptor such as FXR could be a therapeutic target to enhance its function in repressing the synthesis of bile acids in cholestasis [135,136,137].

Finally, intensive research is necessary to further illustrate the signaling pathways and mechanisms of action of different forms of bile acids upon binding to their receptors. There might be novel receptors and novel target genes that could be discovered to be induced or repressed by bile acids. For instance, the implication of CYP3A4 stimulation in cholestasis could provide a novel therapeutic strategy. The expression of CYP3A4 and CYP3A11 is increased in response to cholestasis. However, the function is hindered by the accumulation of bile acids due to its detergent-like action. Genetic variations in the CYP3A4 could enhance CYP3A4 activity in cholestasis and increase detoxification [138,139,140]. UDCA could induce CYP3A4 in mouse models; however, there are discrepancies in the results, and one study reported that it has a minimal effect in humans compared to rifampicin (comprehensively reviewed in [139]). The disruptions in crucial enzymes involved in the synthesis of bile acids, such as CYP7A1 and CYP8B1, can result in variations in the composition of bile acids and impair the absorption of lipids [141]. Additionally, applying breakthrough biotechnological techniques to determine the association of bile acids target genes and some liver diseases using CRISPR-Cas could be beneficial in further understanding the role of nuclear receptors in bile acid homeostasis [142].

## 4. CYP450 Enzymes and Vitamin D

The activation pathway of vitamin D comprises a sequence of sterol hydroxylases that include some of the CYP450 enzymes involved in forming the active hormone, 1α,25-dihydroxyvitamin D3, which acts as a ligand for the vitamin D receptor. First, a hydroxylation step takes place at C25 in the liver, resulting in the production of 25-hydroxyvitamin D. The second step involves 1α-hydroxylation in kidneys, leading to the production of the hormonal variant, 1α,25-dihydroxyvitamin D3 (1,25-(OH)2D3) [142,143].

### 4.1. Role of CYP450 Enzymes in Vitamin D Metabolism

CYP450s participate in the 25- and 1α-hydroxylation processes, as well as the 24- and 23-hydroxylation processes. These processes are thought to trigger the deactivation of the vitamin D molecule [143]. Two hepatic CYP450 enzymes catalyze the 25-hydroxylation of vitamin D3—one is in the mitochondria and the other in the microsomes [143,144]. An enzyme known as mitochondrial vitamin D3 25-hydroxylase appears to be the same as CYP27A, a necessary enzyme for producing bile acid in the liver [145].

#### 4.1.1. CYP27B1 and Vitamin D Activation

CYP27B1, which is also referred to as 1-alpha-hydroxylase, is responsible for the second activation step. It transforms 25-OH-D into 1,25-(OH)2D, known as calcitriol, which is the active form of vitamin D [146]. This enzyme is tightly regulated and has a significant role in preserving calcium homeostasis in the body [147]. Not only is CYP27B1 expressed in kidneys, but it is also expressed in other tissues. For example, an increase in its expression has been observed in muscle fibers affected by denervating diseases, such as amyotrophic lateral sclerosis (ALS), compared to healthy controls [146]. This implies that CYP27B1 could potentially play a part in the signaling of vitamin D under both normal and disease conditions [148].

#### 4.1.2. CYP24A1 and the Inactivation of Vitamin D

CYP24A1, also known as 25-hydroxyvitamin D-24-hydroxylase, plays a role in the deactivation of vitamin D [148]. This enzyme is responsible for transforming 25-OH-D and 1,25-(OH)2D into 24-hydroxylated derivatives, considered an initial step in the breakdown of the vitamin D molecule [149,150]. CYP24A1 is crucial in regulating the concentrations of 1,25-(OH)2D within various tissues [151]. It has been noted that an increased expression of CYP24A1 results in the breakdown of 1,25-(OH)2D, thereby restricting vitamin D signaling within cells [152]. This observation has potential implications for various health issues, including autoimmune disorders such as systemic lupus erythematosus (SLE), where the status of vitamin D and the role of CYP24A1 might be interconnected [151].

#### 4.1.3. Role of Additional CYP450 Enzymes in Vitamin D Metabolism

Several additional CYP450 enzymes, including CYP2R1, CYP3A4, and CYP2D25, can perform 25-hydroxylation on vitamin D [143]. CYP2R1 is the primary hydroxylase enzyme responsible for activating vitamin D by facilitating the production of 25-hydroxyvitamin D (25(OH)D) [153]. Several CYP450 enzymes catalyze various hydroxylation processes for the bioactivation and inactivation of vitamin D3. These processes are vital for vitamin D signaling, regulation, and function [154].

## 5. CYP450 Enzymes as Central Players in Disease

### 5.1. CYP450 and Endocrine Disorders

The body’s production and regulation of steroid hormones, including cortisol, aldosterone, and sex hormones, are indispensable for numerous physiological functions. However, any interference with these processes can result in endocrine disorders that can have severe health implications [153].

#### 5.1.1. PCOS

CYP11A is expressed explicitly in ovulatory follicles’ theca interna and granulosa cells. Studies have shown that a pentanucleotide repeat polymorphism (TTTTA)n in the 5′ untranslated region (UTR) of the *CYP11A* gene is associated with hirsute PCOS patients [155]. The association between these repeat alleles and PCOS susceptibility varies among ethnic groups. A meta-analysis conducted in the Caucasian population revealed a significant connection between the microsatellite (TTTTA)n repeat polymorphism of CYP11A and an increased risk of PCOS [155]. Furthermore, allelic variants of CYP11A and its polymorphisms have been associated with elevated serum testosterone levels, suggesting a potential role in hyperandrogenemia [155]. However, the association between this polymorphism and PCOS differs among populations. Some studies have reported a link between the (TTTTA)n repeat polymorphism in the CYP11A promoter and PCOS in populations from the United States, South India, and Greece. At the same time, no association was found in populations from Spain, China, Argentina, and India [155].

Meta-analysis findings support a connection between PCOS and a pentanucleotide repeat polymorphism in the promoter of the *CYP11A1* gene. Additionally, the association of *CYP11A* with hirsutism, a common symptom of PCOS, suggests that this gene may primarily contribute to hirsutism rather than affecting ovulatory function [155]. In addition, *CYP17* T/C (rs74357) gene polymorphism contributes to PCOS. Susceptibility studies have found that the CC genotype and C allele of the *CYP17* T/C gene polymorphism were associated with an increased risk of PCOS in women. The correlation was robust in Asian populations but not in Caucasians. The study also suggested that obesity may influence the relationship between the *CYP17* gene polymorphism and PCOS [156]. In 2018, two studies found a significant association between CYP17A1 and PCOS in different populations. One study analyzed the rs743572 allele in CYP17A1 in 500 women and found a substantial difference between the PCOS and control groups [157].

Certain polymorphisms in the *CYP19A1* gene have been linked to an increased risk of (PCOS), infertility, and reproductive cancers such as breast, endometrial, and ovarian cancers. These gene variants can disrupt steroidogenesis pathways and contribute to infertility in women with PCOS [155]. Geography and ethnicity may contribute to differences in these associations [155]. In India, a study was conducted involving 394 PCOS cases and 306 healthy women as controls. The researchers found significant differences in the genotypic and allele frequencies of the rs2414096 polymorphism between the PCOS and control groups. They also observed significant associations between the genotypes of the polymorphism and various clinical and biochemical parameters related to PCOS and hyperandrogenism [156]. The results of the first study conducted on PCOS genotypes in Pakistan suggest that the CYP 17 5′-UTR MspA1 (rs743572) (genotype TC) and *CYP19* gene (rs2414096) (genotype GA) polymorphisms are significantly associated with the vulnerability to PCOS in Pakistani women with the traits of infertility and a family history of hypertension [157]. In Egypt, the study concluded that CYP19 rs2414096 polymorphism is associated with aromatase deficiency or reduced aromatase activity with subsequent hyperandrogenism in PCOS Egyptian women [158].

#### 5.1.2. Adrenal Insufficiency

Lack of CYP11A1 activity leads to congenital adrenal insufficiency with 46, XY sex reversal, a lethal endocrine disorder without hormone therapy. Variations in the CYP11A1 gene include nucleotide insertions/deletions in exons/introns and missense mutations. Seven missense mutations and three nucleotide insertions/deletions are identified. For example, an A189V mutation creates a novel splice site, leading to truncated and inactive protein [159]. The L141W mutation is likely to disrupt cholesterol binding. These variations can reduce cholesterol side chain cleavage activity, leading to severe endocrine manifestations in affected individuals [160].

CYP21A2, or steroid 21-hydroxylase, is an enzyme primarily expressed in the adrenal cortex. It is crucial in synthesizing aldosterone from progesterone and cortisol from 17-hydroxyprogesterone [161]. Congenital adrenal hyperplasia (CAH) is caused by mutations in the *CYP21A2* gene, which leads to reduced enzymatic activity in the steroidogenesis pathway. CAH has different forms, with CYP21A2 deficiency being the most common and severe [161]. The nonclassical form is mild and more common, but many mutations remain undiscovered. CAH symptoms vary depending on the extent of enzymatic activity loss and can result in impaired fertility, ambiguous genitalia, and elevated androgen levels. The bovine CYP21A2 structure has provided insights into the effects of mutations, which can affect specific aspects of the enzyme and lead to different forms of the disorder [161].

### 5.2. CYP450 and Liver Disease

Globally, liver disease accounts for approximately two million deaths annually. Cirrhosis accounts for around half of these deaths, with hepatitis and HCC accounting for the remaining 50% [162]. The burden of liver disease varies considerably depending on gender, ethnicity, socioeconomic status, and geographic location and is expected to increase globally [163]. Because CYP450 enzymes are predominantly found in the liver, liver disease modulates their metabolic capacity in complex and unpredictable patterns, suggesting that recommended drug regimens may not be effective in this patient population and supporting the clinical implementation of personalized medicine approaches [164].

#### 5.2.1. Nonalcoholic Fatty Liver Disease

Patients with diabetes, hypertension, and hypercholesterolemia are more likely to develop nonalcoholic fatty liver disease (NAFLD), which is characterized by lipid accumulation in hepatocytes greater than 5% in patients without significant alcohol intake [165]. The different mechanisms of lipid accumulation in the liver include elevated uptake and de novo synthesis of fatty acids and reduced hepatic lipid export and beta-oxidation [166]. NAFLD and its more advanced form of nonalcoholic steatohepatitis (NASH) are increasingly being considered leading causes of cirrhosis and HCC [167].

In 2018, Jamwal et al. investigated the correlation between CYP3A4 activity and NAFLD in 74 human liver tissues from brain-dead donors. Midazolam metabolism rate was used to measure CYP3A4 metabolic capacity. The authors reported reduced CYP3A4 activity and protein expression in NAFLD patients [168]. Further reduction was observed in patients suffering from NASH. Moreover, a decrease in CYP3A4 activity and protein expression associated with diabetes was observed [168]. Their results agree with those of Woolsey et al., who additionally reported that CYP3A4 mRNA levels significantly decreased in NASH patients compared with healthy controls [169]. However, previous genome-wide association studies reported no significant change in the mRNA expression levels of drug-metabolizing genes in NAFLD patients [170,171].

CYP3A4 association with NAFLD was also studied by Powell et al. They performed RNA-seq analysis for 93 liver tissue samples and found that CYP3A4 expression was not significantly downregulated in NAFLD, NASH, and fibrosis samples. CYP2C19 expression showed the most significant downregulation among all three diseases studied. The authors validated their findings by conducting a meta-analysis using 16 studies that reported the association between CYP2C19 and NAFLD, which revealed that CYP2C19 was found to be significantly downregulated in 15 of the 16 studies [172]. This suggests that a personalized medicine approach should be developed for patients taking drugs that are metabolized by CYP2C19, especially those that have a narrow therapeutic window. Additionally, the authors noted that CYP2C19 mRNA levels may not reflect its protein levels, pointing to that as one of the study’s limitations [172]. In contrast, Fisher et al. reported that CYP2C19 downregulated mRNA levels corresponded to decreased protein levels in NASH patients [173].

Numerous studies have demonstrated that CYP2E1 plays an essential role in the progression of NAFLD due to its ability to induce excessive lipid accumulation in the liver cells and promote oxidative stress and inflammation [174]. A novel USP14-HSP90AA1-CYP2E1 axis contributing to NAFLD pathogenesis was reported by Wei et al. In vitro and in vivo data show that ubiquitin-specific proteinase 14 overexpression inhibits the degradation of heat shock protein 90 alpha family class A member 1, resulting in increased CYP2E1 protein levels [175]. No effective NAFLD treatments have been identified due to the complexity of the disease; however, flavonoids are promising therapeutic options based on their ability to regulate CYP2E1 activities [176]. EGCG (epigallocatechin gallate) was reported to have a dose-dependent protective effect against the disease due to its ability to inhibit reactive oxygen species production and CYP2E1-induced oxidative stress in vitro [177]. Another flavonoid, 5-methoxyflavone, was found to alleviate NAFLD through inhibiting the expression of CYP1A1. Using molecular docking techniques, Zhang et al. [178] identified CYP1A1 as a potential 5-methoxyflavone target. This was validated in HepG2 cells by conducting the cellular thermal shift assay (CETSA). Additionally, the authors report that treatment with 5-methoxyflavone reduced the body weight and fat accumulation of high-fat diet-fed C57BL/6J mice by inducing lipase activity. A significant decrease in reactive oxygen species and mRNA levels of inflammation markers such as *TNF-α*, *IL-6*, and *IL-1β* were also observed [178].

#### 5.2.2. Cirrhosis

Fibrosis is the precursor of liver cirrhosis, and cirrhosis is the end-stage condition of chronic liver disease. Due to its asymptomatic nature, patients are usually diagnosed when the disease worsens. However, if detected early, cirrhosis may be reversible [179]. In Japan, Europe, and the United States, alcohol and HCV are the leading causes of cirrhosis. In Asia and Africa, HBV is the leading cause of cirrhosis [180].

Chemical induction is one of the modalities used to mimic the histological pattern of cirrhosis in animal models [181]. Thioacetamide is an organosulfur compound commonly used as an inducer of cirrhosis due to its high reproducibility and low toxicity [182]. These animal models are utilized to study the expression and activity of different CYP450s. However, recent findings suggest that thioacetamide can directly inhibit CYP450 expression independently of cirrhosis [183]. Chandrashekar et al. examined the effect of a 10-week intraperitoneal thioacetamide-induced cirrhosis in male Sprague-Dawley rats on the CYP450 enzymes following a 10-day washout interval to remove the chemical and its direct impact on the enzymes. They reported that CYP2D and CYP3A protein expression decreased by 70% and 30%, respectively, whereas CYP2E1 protein expression remained unaffected. Their results are in agreement with a previous study reporting that cirrhosis has a differential effect on the expression of CYP450, suggesting that their model incorporating the 10-day washout approach produces results that are independent of the direct inhibitory effect of thioacetamide [184,185].

Only two studies have utilized the cocktail approach to generate unbiased estimates of multiple CYP450 activities when administered simultaneously. Frye et al. were the first to adopt the strategy. A cocktail of four oral drugs, caffeine (CYP1A2), chlorzoxazone (CYP2E1), debrisoquin (CYP2D6), and mephenytoin (CYP2C19), were administered to 20 patients with different severity of liver disease and 20 health-matched controls. CYP2C19 was the most sensitive to the presence of liver disease, as measured by the 80% decrease in mephenytoin metabolism in cirrhotic patients [186]. Caffeine, chlorzoxazone, and debrisoquin metabolism decreased by 69%, 60%, and 71% in cirrhotic patients. These results suggested that human liver cirrhosis may selectively affect CYP450 enzyme expression [186].

Duthaler et al. also utilized the cocktail approach to investigate the pharmacokinetics of six drugs and their CYP450-specific metabolites in 36 cirrhosis patients and 12 healthy matched controls in a single-center clinical study (ClinicalTrials.gov, ID: NCT03337945) [187]. Excessive caffeine ingestion and intake of drugs that interact with the cocktail were the exclusion criteria. At the administered doses, no drug–drug interactions were observed. The authors of this study also reported a selective effect on the CYP450 expression profile in cirrhotic patients depending on the severity of the disease and the CYP450 isoenzyme. No change in CYP2C9 expression was observed. However, CYP2C19 expression was decreased in Child A cirrhosis patients, and Child C patients had decreased CYP1A2, 3A, 2B6, and 2D6 levels. Caffeine (CYP1A2), midazolam (CYP3A), omeprazole (CYP2C19), and metoprolol (CYP2D6) elimination rates were significantly decreased by 51%, 60%, 75%, and 37%, respectively. Flurbiprofen (CYP2C9) and efavirenz (CYP2B6) rates were not significantly affected [187].

CYP450 polymorphisms are implicated in liver cirrhosis pathogenesis. In Brazil, a 2004 study investigated the genetic polymorphisms in DNA extracted from the blood samples of 120 alcoholics and 221 healthy controls with a similar ethnic background using the PCR-RFLP technique. Results revealed that the m2/m2 CYP1A1 genotype makes individuals more susceptible to developing liver cirrhosis [188]. Similar methods were employed in a 2016 study in Egypt. However, carriers of the CYP1A1 variants m1 and m3 were not more prone to developing liver cirrhosis or HCC [189].

## 6. CYP450 and Cancer

**Figure 1:**
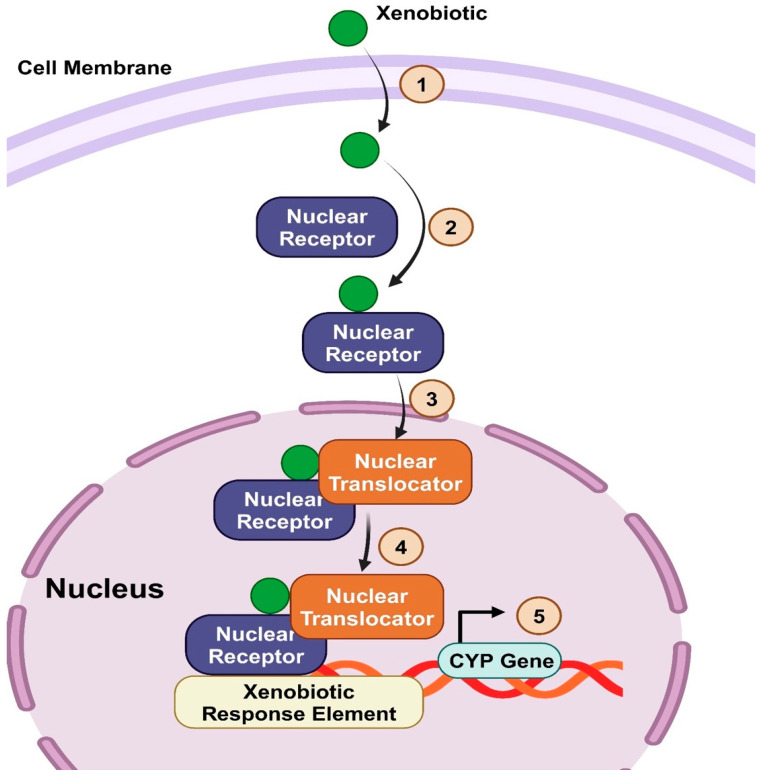
Xenobiotics activate CYP450 gene expression. (1) Xenobiotics translocate to inside the cell. (2) It binds to the NR. (3) Xenobiotic binding induces conformational changes that facilitate its translocation into the nucleus through binding to nuclear translocator. (4) The ligand-bound NR–partner complex nuclear translocator binds to xenobiotic response elements. (5) Initiates the transcription of CYP genes, including those encoding CYP450 enzymes, in various tissues. Created with https://www.biorender.com/ (accessed on 5 June 2024).

In addition to their assistance in activating and deactivating both endogenous and exogenous molecules, CYP450 enzymes are responsible for 66% of the metabolic reactions of carcinogenic agents, as well as their ability to metabolize some compounds, producing carcinogenic metabolites through a “lethal synthesis approach” [11,21]. Some of these compounds are pro-carcinogens because they produce metabolites that can damage DNA. Specifically, CYP1A1, CYP1A2, CYP1B1, CYP2A6, and CYP2E1, as well as their polymorphisms, increase the incidence of some malignancies [190,191,192]. Furthermore, CYP450s have a role in tumorigenesis and chemotherapeutic medicines’ activation and/or metabolism [190]. Changes in the pharmacological effects of drugs in medical conditions like inflammation and the pharmacokinetics of prescribed treatments collectively contribute to CYP450-mediated cancers [193]. Stimulating nuclear receptors such as PXR, aryl hydrocarbon receptor (AhR), and CAR receptors regulate the transcription of CYP450 genes. Xenobiotics, environmental, and behavioral variables stimulate these receptors, as illustrated in Figure 1 [194].

### 6.1. Mechanisms of CYP450-Inducing Cancers

The intricate interaction between genes in carcinogen metabolism makes elucidating a robust predictive model for cancer risk solely based on CYP modulation difficult [190]. The association between CYP450 polymorphism and carcinogenesis is well-studied in the literature [190,192,195,196,197,198,199,200,201,202,203]. AhRs, a ligand-activated transcriptional factor that binds to the Aryl hydrocarbon receptor nuclear translocator (ARNT), initiates the expression of downstream CYP1 family genes, e.g., CYP1A1, CYP1A2, and CYP1B1 [190,192,204]. The nuclear-combined entity then attaches to xenobiotic response elements (XRE). In various malignancies, estradiol and xenobiotics can affect CYP1 expression [190,205].

For instance, CYP1B1, which is highly expressed in many cancers, converts estradiol to 4-hydroxyestradiol, which is oxidized by peroxidases into the probable carcinogenic metabolite estradiol-3,4-quinones [190,206,207]. Furthermore, CYP1B1 can transform the heterocyclic amine 2-amino-1-methyl-6-phenylimidazole [4,5-b] pyridine contained in foods into N2-hydroxylated derivatives, which have DNA genetic modification characteristics that are believed to be associated with the prevalence of cancers of the colon and breast. This enzyme is active in tumors and may metabolize several anticancer medicines [204]. Also, polycyclic aromatic hydrocarbons are well-known carcinogens that have the potential to cause tumors when metabolized by CYP450s. They are metabolized and stimulated by CYP1A1 and CYP2W1 to form positively charged epoxy compounds with high carcinogenic activity. CYP2W1 is a monooxygenase enzyme significantly expressed in tumor tissues and fetal development [204]. This step alters DNA base pairing by causing codon alterations, generating genetic changes, and finally leading to cancer [207].

The human CYP4 family plays a crucial role in tumor formation. CYP4A11, CYP4F2, and CYP4F3B hydroxylate arachidonic acid at its omega position, resulting in hydroxyeicosatetraenoic acid (20-HETE) production, which is the primary pro-inflammatory metabolite that affects vascular remodeling and affects the development of new blood vessels under hypoxic conditions. This compound significantly affects tumor growth and angiogenesis [190,192,207,208,209]. CYP4B1 is highly expressed in bladder and urinary tract carcinoma, breast carcinoma, ovarian carcinoma, lung cancer, and prostate cancer [210].

Tobacco smoke’s nitrosamines are believed to contribute to lung cancer. In the lungs, the predominant nitrosamine transformation is handled by CYP2A13, which produces alkyl-diazohydroxides. These harmful byproducts alkylate DNA bases, producing methylated bases such as O6-methylguanine, 7-methylguanine, and O4-methyl thymidine [211]. Nitrosamines can impede the process of DNA repair by the enzyme O6-methylguanine methyltransferase, resulting in the accumulation of methylated DNA bases. It was reported that there is a significant correlation between alkylated DNA bases and tumorigenesis. Individuals with a mutant allele of the nitrosamine metabolizing CYP2A13 with reduced enzymatic activity due to the amino acid shift R257C had a decreased risk of lung cancer, particularly adenocarcinoma lung cancer [211]. Finally, CYP450s are involved in approximately all cancer types, especially the CYP1 family. No specific CYP450 enzyme or polymorphism contributes to a particular type of cancer. CYP1B1 and CYP4B1 are involved in the tumorigenicity of different cancers, like ovarian, breast, and prostate [207,210].

### 6.2. CYP450 and Chemotherapy

The CYP450 enzymes are responsible for the metabolism of numerous anticancer medicines. Variability in CYP450 enzyme expression can impact the effectiveness of anticancer drugs. This is particularly relevant for traditional chemotherapy targeting tumors and healthy cells [190]. Variations in drug-metabolizing enzymes, specifically CYP2B6, CYP2C9, CYP2C19, CYP2D6, CYP3A4, and CYP3A5, can lead to unsuccessful therapeutic or adverse effects [190,191].

The primary adverse effect of the doxorubicin and cyclophosphamide (AC) regimen is neutropenia. CYP2B6 polymorphisms have been linked to worsening stage 4 neutropenia in individuals undergoing the AC regimen [190]. CYP1 family expression can modulate phytoestrogens and dexamethasone, potentially compromising cancer treatment [190,205]. The increased expression of CYP1B1 causes resistance to docetaxel, which can be restored using a CYP1B1 inhibitor by inhibiting its enzymatic activity [190]. In addition, CYP1B1 increases resistance to several chemotherapeutic drugs, such as Tamoxifen and cisplatin [212]. Tamoxifen is an endocrine therapy used in pre- and post-menopausal women who are unable to tolerate aromatase inhibitors [213]. Tamoxifen, a prodrug, is metabolized by CYP2D6, CYP3A4, CYP2B6, and CYP2C19. In some cases, CYP2D6 might exhibit poor enzymatic activity, resulting in inadequate activation of Tamoxifen and subsequent treatment failure [190].

For other drugs like vincristine, biotransformation is substantially greater with CYP3A5 than with CYP3A4. Children with precursor B cell acute lymphoblastic leukemia who express CYP3A5 had reduced vincristine-induced neuropathy in their extremities and generated more primary metabolites than those who did not express the enzyme. The CYP3A5 genotype significantly impacts vincristine excretion and exposure for patients [190]. The *CYP3A5* gene is a robust predictor of vincristine toxicity, perhaps leading to improved dosage regimens for a medication used to treat numerous curable children’s malignancies [190]. Increased CYP450 isoforms CYP3A4 and CYP2C8 levels in cancerous breast tissue may restrict cytosolic concentrations of taxanes such as paclitaxel (PTX) and contribute to treatment resistance. This restriction leads to an inability to respond effectively to taxane treatment, indicating the significance of CYP3A4′s presence in cancerous breast tissue. CYP3A4 and CYP2C8 convert PTX to the inactive metabolites 3′-para-hydroxy-PTX (3′-p-OH-PTX) and 6α-hydroxy-PTX (6-OH-PTX), respectively [214].

Hence, the role of CYP450s in the activation and deactivation of chemotherapeutic agents through the metabolism process needs more investigation.

### 6.3. BC

Estrogen-dependent cancer refers to a type of cancer that is influenced by estrogen. Estrogen-dependent cancers are sub-categorized into breast, ovarian, and endometrial cancer (EC) [82]. Estrogen is a vital hormone in the female body, regulating various physiological processes, including the growth and development of reproductive tissues. Estrogen receptors on the cancer cells allow estrogen to bind to these receptors, triggering cellular signaling pathways that promote cell growth and division. This interaction between estrogen and its receptors can fuel the growth of cancer cells and contribute to tumor development [82].

Several CYP450 genes play a role in the production and breakdown of estrogen, such as the CYP1B1 enzyme, which metabolizes estradiol to 4-hydroxyestradiol. This metabolite can form harmful free radicals, damaging DNA and carcinogenesis [215]. The risk of breast cancer can be increased by the CYP450 genes involved in estrogen synthesis and metabolism [216]. CYP19A1 genetic variants are associated with elevated levels of circulating estrogen hormone, mainly in postmenopausal women. Likewise, women with high body mass index (BMI) show higher CYP19A1 mRNA gene expression [217]. Studies have shown that high levels of CYP19A1 mRNA are associated with local recurrence and incidence of metastases, as well as death due to BC [218]. On the other hand, it has been observed that in premenopausal patients, higher levels of circulating estrogen may be associated with lower expression of the CYP19A1 gene, which can result in increased recurrence rates. This suggests an inverse correlation between CYP19A1 mRNA levels and high estrogen levels in such patients [218].

CYP1A1 is associated with the aggressiveness of BC. The *CYP1A1* gene is crucial in metabolizing various compounds, including polycyclic aromatic hydrocarbons (PAHs) and estrogens. Previous research has investigated the link between specific variations in the *CYP1A1* gene and higher rates of cancer development of cancer, especially BC, in Caucasian populations. CYP1A1 was found to be expressed in about 90% of breast tumors, but the level of expression varied among tumors. It did not correlate with estrogen receptor alpha levels, tumor grade, or clinical outcomes [219]. Four common *CYP1A1* gene polymorphisms have been identified: CYP1A1*2A, CYP1A1*2C, CYP1A1*3 (specific to African Americans), and CYP1A1*4. Epidemiological studies examining the association between these *CYP1A1* gene polymorphisms and BC risk have produced conflicting results, with some studies showing a significant association and others showing no association [220]. The distribution of CYP1A1*2A and CYP1A1*2C genotypes vary among different populations, with higher frequencies observed in Asians than Caucasians and African Americans. In a Chinese population, having both CYP1A1*2A and CYP1A1*2C polymorphisms together was linked with a decreased risk of BC, particularly among postmenopausal women with lower BMI or women with a low waist-to-hip ratio [221]. CYP1A1 is linked to the 2-hydroxylation of estrogens, and the connection between CYP1A1 genotypes and BC risk could be affected by estrogen levels. The theory is that high CYP1A1 activity coupled with high estrogen levels could increase risk, while high CYP1A1 activity with low estrogen levels could decrease the risk [218]. Furthermore, a study conducted on the Jordanian population showed that certain genetic variations in CYP450 genes were associated with hormone receptor status in BC patients. These findings contribute to our understanding of the genetic factors influencing BC susceptibility and prognosis in the Jordanian population, potentially paving the way for personalized approaches to BC prevention and treatment. Specific CYP450 gene polymorphisms were associated with prognostic factors in BC (Table 3), such as tumor stage, hormone receptor status (estrogen and progesterone receptors), and human epidermal growth factor receptor 2 (HER2) status. They aimed to determine if specific genetic variations influenced the clinical characteristics and outcomes of BC patients in the Jordanian population [220].

The study findings revealed significant associations between specific CYP450 gene polymorphisms and breast cancer risk in the Jordanian population [220]. Specific genetic variations in CYP1A1 and CYP1B1 genes were found to be more prevalent in certain cancer patients compared with the control group, suggesting that these polymorphisms might contribute to an increased susceptibility to cancer [222,223,224]. Regarding prognostic factors, the researchers observed associations between CYP450 gene polymorphisms and hormone receptor status in BC patients. Certain genetic variations in CYP1A1 and CYP3A4 genes were linked to hormone receptor-positive BC, indicating a potential role of these polymorphisms in hormone receptor expression and hormonal signaling pathways [220].

### 6.4. Ovarian and EC

Different CYP1B1 variants are associated with variability in the therapeutic response to ovarian cancer treatment and immune checkpoint inhibitors. The Ile462Val variant increases the risk of ovarian cancer in Caucasian people. Another variant called Thr461Asn has a weaker association with ovarian cancer risk. Moreover, the rs2470893 variant is associated with an increased risk of both ovarian and EC in people carrying the AA allele [221].

Concerning EC, polymorphisms of the *CYP1B1* gene and the activation of estrogen receptors significantly contribute to the pathogenesis of EC [225]. The L432V polymorphism of the *CYP1B1* gene and its effect on the expressions of estrogen α and β (ERα and ERβ) were investigated, and the study found a significant difference in the genotypic distributions and allelic frequencies of the codon 432 regions of CYP1B1 between EC patients and healthy controls in a Datong (China) population [226]. A mutant G allele revealed a 2.213-fold higher risk of having EC than allele C. The relative risk was calculated at 3.235-fold compared with wild-type C/C. The higher frequency of patients with G/G genotype or G allele indicates that a person carrying this genotype or allele has an increased risk. The study suggests that the mutant genotype of CYP1B1 might be a risk factor for EC [226,227]. The Ile/Val EC (CYP1A1*2C) polymorphism in Japan was higher in EC. Moreover, the Val/Val genotype was reported to be more frequent in the EC group than in the control subjects. However, there was no significant association between gene polymorphisms and pathological features of gynecological malignancies [228,229]. The overrepresentation of the CYP1A1*4 allele in both EC and endometrial hyperplasia suggests that the CYP1A1*2C genotype had a 5-fold higher risk of developing endometrial hyperplasia than those with Ile/Ile. The CYP1A1 Ile/Val allele was also more frequent in the EC group. The CYP1A1*4 allele may increase the relative risk of developing this cancer [225]. The relationship between gene polymorphisms in CYP1A1 and CYP1B1 and the risk of developing type I EC in women was investigated in China, and the study found that a specific polymorphism (rs4646421) in the *CYP1A1* gene significantly differed between patients with type I EC and control subjects. Individuals with the CC genotype had a lower risk of EC compared with those with the CT genotype [225]. Additionally, a specific allele (T) was associated with a higher risk of EC. The study also found associations between specific genotypes and risk factors such as hypertension, age, BMI, and menopausal delay. However, no significant difference was observed in the frequency of a polymorphism (rs1056836) in the *CYP1B1* gene between the two groups. The findings suggest that the rs4646421 polymorphism in the *CYP1A1* gene may increase the risk of type I EC and could serve as a potential biomarker for screening this type [225].

### 6.5. Hepatocellular Carcinoma

HCC, the most common form of liver cancer, is the fifth most common cancer in men and the ninth in women. It is the third leading cause of death from cancer globally [230]. Studies reporting how HCC affects drug-metabolizing enzymes are limited despite the liver being the primary site of biotransformation. To investigate whether CYP450 enzyme activity was affected in HCC patients, Zhou et al. [231] studied the expression of ten major CYP450s’ in 102 HCC liver tissue samples and 105 non-cancerous tissue. They report that the intrinsic clearance of CYP1A2, CYP2C19, and CYP2C8 decreased. CYP2E1, CYP2D6, and CYP2C9 showed increased intrinsic clearance, while no change was observed in CYP3A4/5, CYP2B6, and CYP2A6. The same changes were observed in HCC patients with fibrosis or cirrhosis, except CYP2D6, which had increased levels in patients with cirrhosis. The authors also report that three polymorphisms, CYP3A5*3, CYP2D6*10, and CYP2C9*3, had pronounced effects on enzyme activity. An interesting finding of this study is that CYP2D6*10 mutant frequency was significantly different between HCC and healthy tissues, indicating its potential as a predictor of HCC carcinogenesis [231].

CYP1A2 is downregulated in 90% of HCC patients [232,233]. Recent findings suggest it suppresses HCC by regulating the signaling pathways of the hepatocyte growth factor (HGF)/mesenchymal-epithelial transition factor (MET) and the Wnt/Dvl2/β-catenin signaling. MET is an oncogene highly expressed in 80% of HCC patients [233]. It is currently the only known receptor of HGF, which, at high levels, is a poor prognostic indicator. The HGF/MET signaling pathway is critical in liver tumor proliferation and metastasis [234]. By decreasing the expression levels of HGF and HIF-1a (a transcriptional activator of MET, CYP1A2) was able to suppress MET activation and inhibit HCC cell viability, clonogenicity, migration, and invasion abilities in vitro and carcinogenesis in vivo [235]. CYP1A2 is also able to suppress HCC tumorigenesis by inhibiting the Wnt/β-catenin signal, reducing Dvl2 (a core component of Wnt signaling) expression by inducing its interaction with an E3 ubiquitin kinase, leading to its degradation, and promoting the accumulation of reactive oxygen species [236].

CYP1A2 was also reported to play a role in overcoming sorafenib resistance in HCC. Sorafenib chemotherapy has been considered the frontline treatment for patients with advanced, unresectable HCC since 2007. However, poor clinical outcomes have been observed due to acquired drug resistance [237]. Yu et al. established a sorafenib-resistant HCC cell line that they followed to have enhanced proliferation, colony formation, and invasion capabilities. CYP1A2 expression was downregulated and inversely correlated with NF-κB (Nuclear Factor Kappa-Light-Chain-Enhancer of Activated B Cells) expression in these cells. Upon overexpression of CYP1A2, sorafenib-resistant cells were found to have reduced NF-κB expression and proliferation rates and enhanced sorafenib sensitivity, whereas CYP1A2 silencing showed opposite effects. The authors also investigated the effects of omeprazole, a CYP1A2 inducer, in combination with sorafenib. They reported a synergistic effect as the tumor growth in vitro and in vivo was significantly hindered [238]. Additionally, CYP1A2 was reported to play a vital role in the gender disparity of HCC by metabolizing 17β-estradiol to generate the potent anti-tumor metabolite 2-methoxy estradiol, which inhibits HCC growth in vitro and in vivo. 17β-estradiol, in combination with sorafenib, showed an additive effect on HCC development [238].

CYP4 family members were found to be involved in the development of different types of cancer, including breast, ovarian, and thyroid cancer [239]. In HCC, evaluation of the CYP4 family expression profile in 377 cases from The Cancer Genome Atlas datasets revealed that high CYP4F12, CYP4V2, and CYP4F2 mRNA expression levels were negatively correlated with cell cycle-related genes and positively correlated with better overall survival, suggesting that these CYP4 genes are favorable prognostic markers in HCC patients [240]. Another study confirmed the upregulation of CYP4A11 in non-cancerous liver tissues compared to HCC tissues through quantitative RT-PCR and Western blotting assays. The authors also reported that CYP4A11 expression is an independent prognostic marker of overall and disease-free survival [241].

The role of chronic inflammation in the progression of HCC remains understudied. AA is a critical inflammatory process regulator by forming mediators such as eicosanoids [242]. In a recent study by Wang et al. [243], they reported that dysregulation of the CYP450 metabolic pathway of AA is implicated in HCC progression and invasion. Publicly available datasets of clinical HCC tissues and tissue microarrays were investigated using bioinformatics tools, proteomics, and immunohistochemistry [243]. The results demonstrated a significant correlation between HCC pathological features and the dysregulation of the AA metabolic pathway. The expression of CYP2C19, CYP2C9, CYP2C18, and CYP2C8 was negatively associated with alpha-fetoprotein, carbohydrate antigen 19-9, alkaline phosphatase, aspartate transaminase, and gamma-glutamyl transpeptidase levels. Histological grade and microvascular invasion were also negatively correlated with the CYP2 family genes. These findings could have critical clinical applications and be utilized as potential markers of early HCC recurrence [243].

## 7. Future Perspectives

Artificial intelligence (AI) may be used to develop new drugs. AI techniques have been widely used in drug metabolism to predict how the body would metabolize medications. Otherwise, using AI allows for rapidly examining massive chemical datasets, providing substantial insight into their potential metabolism. AI systems can simultaneously detect metabolic interactions between many compounds, aiding drug development [244,245]. In addition, AI techniques can provide considerable data on potential metabolic pathways, saving valuable resources for in vitro and in vivo research. Indeed, AI techniques in medical research and development can enhance pharmaceutical safety and efficacy by creating new compounds with enhanced metabolism [244]. Identifying the inhibitors of CYP450 family members may aid in the elimination of poorly functioning medications during the preliminary phase of the drug discovery procedure, reducing the possibility of stopping pharmaceutical production, discontinuing drug usage, withdrawing it from the market, or limiting its usage, all of which are critical to the development of new drugs [246]. Therefore, future research should focus more on developing and testing algorithms to anticipate enzyme substrates, how the drug will be metabolized, and discovering inducers and inhibitors for each enzyme. In addition to conducting experimental studies on the functions of various CYP450 genes and polymorphisms, the outcomes of these studies could enhance and encourage the personalized medicine field.

## 8. Concluding Remarks

CYP450 enzymes represent a diverse superfamily that is crucially involved in metabolizing xenobiotic and endogenous compounds. Their primary function lies in Phase I biotransformation, where they catalyze approximately 80% of the oxidative metabolic reactions of a wide range of substrates, including hormones, lipids, and xenobiotics. This activity significantly influences the pharmacological efficacy and elimination of drugs and the physiological roles of endogenous compounds. Beyond drug metabolism, CYP450 enzymes are essential for maintaining physiological homeostasis. They participate in metabolizing endogenous substances. These enzymes are inducible and inheritable, with several polymorphisms for each family member. The biosynthesis and metabolism of steroid hormones rely on specific classes of enzymes, including CYP450 enzymes. These enzymes are crucial in maintaining hormonal balance within the body.

They also catalyze fatty acid oxidation, cholesterol metabolism, and bile acid biosynthesis. Members of the CYP450 families have diversity in their structure and function, which eventually enables them to contribute to regulating cellular lipid levels and metabolic fluxes. So, various metabolic disorders have been linked to a dysregulation of CYP450-mediated lipid metabolism. CYP450 isoforms also contribute to synthesizing specific endogenous compounds, including potentially toxic metabolites. Notably, CYP7A1 and CYP3A4 demonstrate involvement in synthesizing and metabolizing bile acids, respectively, highlighting the interaction between these enzymes and some nuclear receptors (FXR/PXR/CAR) in maintaining homeostasis. The diverse functionality of CYP450 enzymes extends to vitamin D metabolism, with enzymes like CYP27B1 facilitating the activation of 1α,25-dihydroxyvitamin D3, essential for calcium balance and bone health. Conversely, some CYP450 enzymes, like CYP1A1, CYP1A2, CYP1B1, CYP2A6, and CYP2E1, have a role in the onset and development of many cancers by converting pro-carcinogenic substrates into carcinogens.

At the same time, the CYP4 family plays a crucial role in tumor formation. The CYP1 family plays a vital role in chemotherapeutic drug metabolism and resistance. In addition, CYP2D6, CYP3A4, CYP2B6, and CYP2C19 are implicated in anti-cancer metabolism, influencing their efficacy. Understanding the dynamic relationship among CYP450 polymorphisms, expression levels, and functional activity provides significant promise for personalized medicine approaches. Finally, researchers can facilitate targeted therapy approaches in clinical practice by clarifying the impact of CYP450 dysregulation in various pathologies, such as endocrine disorders, hypercholesterolemia, certain cancers, and liver diseases.

## Figures and Tables

**Table 1:** CYP450 families, main functions, and substrates [15,39].

CYP Family	Main Function	Subfamily	Substrate/Endogenous Compound
CYP1	Biotransformation	CYP1A1	Granisetron and riociguat
		CYP1A2	Polycyclic aromatic hydrocarbons, caffeine, tizanidine, clozapine, olanzapine, theophylline, alosetron, duloxetine, melatonin, pirfenidone, ramelteon, tasimelteon, acetaminophen, antipyrine, bufuralol, ondansetron, phenacetin, and tacrine
CYP2	Biotransformation	CYP2A6	Nicotine, coumarin, and valproic acid
		CYP2B6	Efavirenz
		CYP2C8	Metabolizes over 60 clinically relevant drugs, including montelukast, pioglitazone, repaglinide, and rosiglitazone
		CYP2C9	Warfarin, carvedilol, celecoxib, glipizide, glimepiride, ibuprofen, irbesartan, losartan, phenytoin, and tolbutamide
		CYP2C19	Omeprazole, lansoprazole, and phenobarbital
		CYP2D6	Antidepressants, antipsychotics, beta-blockers, antiretroviral agents, antiarrhythmics, morphine derivatives, and Tamoxifen
		CYP2E1	Ethanol, acetaminophen, theophylline, and verapamil metabolism
CYP3	Biotransformation	CYP3A4	Alprazolam, amlodipine, buspirone, calcium channel blockers, caffeine, citalopram, clopidogrel, cocaine, cyclosporine, diazepam, erythromycin, drugs, montelukast, quetiapine, sertraline, sildenafil, statin drugs, tacrolimus, warfarin, zolpidem, estradiol, lidocaine, losartan, and many chemotherapeutic agents
CYP4	Fatty acid metabolism	CYP4A	Arachidonic acid (AA)
CYP5	Thromboxane A2 synthesis		-
CYP7	Bile acid biosynthesis	CYP7A1	Converts cholesterol to 7α-hydroxycholesterol
		CYP7B1	Metabolizes dehydroepiandrosterone
CYP8	Bile acid and prostacyclin biosynthesis	CYP8A1	Converts prostaglandin H_2_ to thromboxane A
CYP11	Steroid biosynthesis	CYP11A1	Converts cholesterol to pregnenolone plus 4-methylpentanal
CYP17	Steroid biosynthesis	CYP17A1	Converts corticosterone to cortisol
CYP19	Steroid biosynthesis	CYP19A1	Metabolizes androstenedione and testosterone
CYP20	Unknown function		-
CYP21	Steroid biosynthesis	CYP21A2	Converts progesterone to deoxycortisone in pregnancy
CYP24	Vitamin D degeneration	CYP24A1	Converts 1,25-dihydroxyvitamin D3 (calcitriol) to 1α,24R,25-trihydroxyvitamin D3
CYP26	Retinoic acid hydroxylation	CYP26A1, CYP26B1, and CYP26C1	Convert retinoic acid to 4-hydroxyretinoic acid
CYP27	Vitamin D3 and bile acid biosynthesis	CYP27C1	Converts retinol (vitamin A1) to 3,4-didehydroretinol (vitamin A2)
CYP39	Cholesterol synthesis	CYP39A1	Converts 24-hydroxycholesterol to 7α,24-dihydroxycholesterol
CYP46	Cholesterol synthesis	CYP46A1	Converts cholesterol to 24(S)-hydroxycholesterol
CYP51	Cholesterol synthesis	CYP51A1	Lanosterol

**Table 2:** CYP450 enzymes, their origin, and role in steroidogenesis [75].

CYP450 Enzyme	Origin	Role
CYP11A1	Mitochondria	Cholesterol side-chain cleavage enzyme- desmolase
CYP17A1	sER (smooth endoplasmic reticulum)	17 alpha-hydroxylase or 17,20-lyase
CYP21A2	sER	21-hydroxylase
CYP11B2	Mitochondria	11beta-hydroxylase
CYP18B2/18-HSD	Mitochondria	Aldosterone synthase
CYP19A1	sER	Aromatase

**Table 3:** CYP 450 polymorphisms and their impact on BC [220].

CYP450 Gene	Variants	Impact
CYP19A1	rs7176005 rs6493497	Significantly associated with BC and creates a variable response to aromatase inhibitors at the initial stages of BC.
	rs700519	Associated with BC at menopause. Significantly associated with age at BC diagnosis and lymph node involvement.
	rs10046 rs4646	The rs4646 conferred a beneficial effect in increasing metastatic time in BC patients.
CYP2C19	rs4244285	Significantly associated with HER2
CYP1A2	CC genotype of rs762551	Protective factor against progression and development. Significantly associated with age menopause, HER2, histology classification, and lymph involvement.

## Abbreviations

AA	Arachidonic acid
ACTH	Adrenocorticotropic hormone
AhR	Aryl hydrocarbon receptor
AI	Artificial intelligence
ALS	Amyotrophic lateral sclerosis
ARNT	Aryl hydrocarbon receptor nuclear translocator
AS-PCR	Allele-specific PCR
BC	Breast cancer
BMI	Body mass index
CA	Cholic acid
CAH	Congenital adrenal hyperplasia
CAR	Constitutive androstane receptor
CDCA	Chenodeoxycholic acid
CETSA	Cellular thermal shift assay
CNVs	Copy number variations
CYP 450	Cytochrome P450
DHA	Docosahexaenoic acid
EC	Endometrial cancer
EETs	Epoxyeicosatrienoic acids
EGCG	Epigallocatechin gallate
EM	Extensive metabolizers
EP	Epoxy
EPA	Eicosapentaenoic acid
EpOMEs	Epoxy Octadecanoic Acids
FDA	Food and Drug Administration
FXR	Farnesoid X receptor
HCC	Hepatocellular carcinoma
HETE	Hydroxyeicosatetraenoic acid
HGF	Hepatocyte growth factor
HNF4	Hepatocyte Nuclear Factor 4 alpha
HSD	Hydroxysteroid dehydrogenase
IMs	Intermediate metabolizers
LA	Linoleic acid
LCA	Lithocholic Acid
LRH-1	Liver Receptor Homolog 1
MET	Mesenchymal-epithelial transition factor
NAFLD	Nonalcoholic fatty liver disease
NASH	Nonalcoholic steatohepatitis
NF-κB	Nuclear Factor Kappa-Light-Chain-Enhancer of Activated B Cells
NMD	Nonsense-mediated mRNA decay
NRs	Nuclear receptors
PBREM	Phenobarbital Responsive Enhancer Module
PCOS	Polycystic ovary syndrome
PCR	Polymerase chain reaction
PMs	Poor metabolizers
PTX	Paclitaxel
PUFAs	Polyunsaturated fatty acids
PXR	Pregnane X Receptor
PXREs	PXR Response Elements
RFLP	Restriction fragment length polymorphism
RT-PCR	Real-time PCR
RXR	Retinoid X Receptor
sER	Smooth endoplasmic reticulum
SHP	Small Heterodimer Partner
SLE	Systemic lupus erythematosus
SNPs	Single nucleotide polymorphisms
StAR	Steroidogenic acute regulatory
STARD3	StAR-related lipid transfer domain-3
UDP	Uridine diphosphate
UM	Ultra-rapid metabolizers
UTR	Untranslated region
VNTRs	Variable number tandem repeats
XREs	Xenobiotic response elements
